# Usefulness of Selected Acute-Phase Proteins in the Postsurgical Monitoring of Arthroscopy and Splint Bone Removal in Horses

**DOI:** 10.3390/ani11102952

**Published:** 2021-10-13

**Authors:** Maciej Przewoźny, Magdalena Senderska-Płonowska, Anna Rząsa, Heliodor Wierzbicki, Jacek Borkowski, Jan-Hein Swagemakers, Agnieszka Żak-Bochenek, Tadeusz Stefaniak

**Affiliations:** 1 Klinika dla Koni Equi Vet Serwis Dr Maciej Przewoźny, 64-320 Buk, Poland; m.przewozny@klinika-konie.com.pl; 2 Department of Immunology, Pathophysiology and Veterinary Preventive Medicine, Wrocław University of Environmental and Life Sciences, 50-137 Wrocław, Poland; anna.rzasa@upwr.edu.pl (A.R.); agnieszka.zak@upwr.edu.pl (A.Ż.-B.); tadeusz.stefaniak@upwr.edu.pl (T.S.); 3 Department of Genetics, University of Environmental and Life Sciences, 50-137 Wrocław, Poland; heliodor.wierzbicki@upwr.edu.pl; 4 Department of Biochemistry, Academy of Physical Education, 50-137 Wrocław, Poland; jacek.borkowski@awf.wroc.pl; 5 Tierärztliche Klinik für Pferde in Lüsche, 49456 Bakum, Germany; jswagemakers@t-online.de

**Keywords:** horse acute phase proteins, fibrinogen, haptoglobin, arthroscopy, splint bone removal

## Abstract

**Simple Summary:**

The study shows the changes in concentration of acute phase proteins (fibrinogen, haptoglobin, protease inhibitors) in the serum of patients from the equine clinic. Blood was collected from horses that underwent arthroscopy (41 horses) and splint bone removal (13 horses). The concentration of the above-mentioned proteins was recorded from the time before the surgery until the 28th day after the surgery and compared to a control group of healthy horses (60 horses). Acute phase proteins change their concentration before clinical symptoms appear; therefore, they could play a key role in early recognition and preventing complications.

**Abstract:**

Background: Arthroscopy and splint bone removal are the common orthopedic procedures in horses. Estimation of the dynamics of acute phase proteins in postoperative monitoring seems to be interesting diagnostic approach. The aim of the study was to investigate changes in the concentrations of plasma inflammatory markers—fibrinogen, haptoglobin, and protease inhibitors—following orthopedic surgery in horses. The study involved 114 horses, divided into two study groups undergoing: arthroscopy (41 horses) and splint bone removal (13 horses). The control group consisted of 60 healthy horses. The blood was collected before the surgery and 24, 48, 72 h, 5, 7, 10, 14 and 28 days after the surgery. Plasma fibrinogen, serum haptoglobin and proteinase inhibitors were measured. Results: In non-complicated cases of arthroscopy and splint bone removal, fibrinogen and haptoglobin increased stepwise from 24 h, achieved the maximum level at 72 h and returned to preoperative levels after 10–14 days. In one complicated case after arthroscopy surgery the marked increase in fibrinogen and haptoglobin concentrations was observed 24 h earlier than standard parameters of inflammation Conclusion: The study shows the evolution of APPs after arthroscopy and splint bone removal in 28 days postsurgery period and in the case of one complicated case of arthroscopy.

## 1. Background

Arthroscopy and splint bone removal are common types of orthopedic surgery in horses. An elevated risk of surgical site infection [1] occurs during arthroscopic removal of large osteochondral fragments and may lead to septic arthritis. The risk of postoperative complications after clean orthopedic surgery [2] as well as joint infection after arthroscopy [3] is comparable when no antimicrobials or pre-/perioperative antimicrobial therapy has been applied. In horses, the APR is manifested in a variety of physiological processes in conditions of excessive stress induced by transport and physical exercise, and this emphasizes the role of APP as an important biomarker in horses [4,5,6].

Measuring serum markers of inflammation may support the clinical assessment of post-operative recovery and provide valuable information on the severity of inflammation earlier than routine inspections every 2–3 days postsurgery [7]. The evaluation of the serum acute phase proteins (APPs) concentrations seems to be of interest in the monitoring of health and welfare in horses, as well as to assess their health status following different surgical treatments. In equine clinics, horses usually receive an intravenous cannula after surgery, so taking blood samples every day is harmless and convenient. Sequential blood sampling may aid in the assessment of the increase and decrease in an inflammatory response. It allows the recognition of excessive inflammation/wound infection earlier than analyses based on clinical signs of post-operative discomfort and pain. Effective monitoring can also limit the use of postoperative antibiotics and non-steroidal anti-inflammatory drugs. 

Fibrinogen (Fb) and haptoglobin (Hp) are slow reacting positive acute phase proteins frequently used in horses [8,9,10,11,12,13,14,15,16]. Fb was assessed as a highly sensitive (82%) marker gsof an inflammatory state in horses [17] and post-operative infections in humans [18] Hp was found in the synovial fluid in arthritic joints in horses [15] and the intraarticular concentration of Hp was found to correlate with its serum levels [19]. Protease activity plays a key role in the loss of the integrity of the cartilage matrix and in the impairment of the equine joint function [20]. Hence, although there is no previous data about the response of protease inhibitors (PI) to surgery in horses, they were included in this study.

The aim of the study was to investigate changes in the concentrations of the plasma inflammatory markers Fb, Hp, and PI following orthopedic surgery in horses. The focus of the study was to assess the relationship between the extent of tissue damage, the clinical severity of inflammation and the dynamics of APPs reaction. To the best of the authors’ knowledge, this is the first study assessing the association between an orthopedic surgery and APPs on a large equine population tested in clinical conditions and the first with a long observation period.

## 2. Methods

### 2.1. Horses

The study was performed as a prospective clinical trial. The research was conducted with the consent of the Second Local Ethics Committee for Experiments on Animals at the Agricultural University of Wroclaw (23.04.2004 r. No. 22/04). The study involved 114 horses, which were divided into two study groups: “group A” undergoing arthroscopy (41 horses) and “group R” undergoing splint bone removal surgery (13 horses). The “control group” of healthy horses included patients of the clinic examined at the routine prophylactic examinations (60 horses). All horses were patients of the Tierärztliche Klinik für Pferde in Lüsche, Germany in 2002–2005. The animals were included in the study with the informed consent of their owners.

In Group A there were 18 mares, 19 geldings and four stallions aged between 2.5 and 10 years (mean: 4.3). In Group R there were seven mares, five geldings and one stallion, aged between 3 and 8 years (mean: 5.15). In both groups all horses were warmblood horses including one thoroughbred and one German Riding Pony. In the control group, there were 20 mares, 20 geldings and 20 stallions, ranging in age between 3 and 17 years (mean: 5.75). There were 31 Oldenburger horses, 14 Hanoverian horses, six Holsteiner horses, four Westphalian horses, two Holsteiner/KWNPx horses and a single thoroughbred horse, a thoroughbred/Arabian mix horse and a Selle France horse. All horses in the control group underwent a clinical and orthopedic examination, which ruled out any abnormalities. 

### 2.2. Clinical Procedures

Horses were assigned to the study groups A and R based on the results of the anamnesis, clinical, orthopedic and radiological examination, which indicated the need for arthroscopic surgery to remove a free osteochondral fragment in the joint space (group A) or splint bone removal surgery (group R). In group A, arthroscopic surgery was performed on one joint in 18 horses, on two joints in 21 horses and on three joints in two horses, and included procedures on the fetlock (37 interventions), hock joint (19 interventions), stifle joint (six interventions), pastern joint (two interventions) and coffin joint (two interventions)—for a total of 66 joints. In all the horses in group R, the surgery consisted of removing a single splint bone that was broken. In six horses, this involved the medial splint bone in the left thoracic limb. The medial splint bone in the right thoracic limb was removed in five horses, while the lateral splint bone in the right pelvic limb was operated on in one horse, and the lateral splint bone in the left pelvic limb was removed in one horse. 

The general anesthetic procedure was the same for all horses. The horses were premedicated using xylazine (0.36 mg/kg b.w, Xylavet 100 mg/mL, CP-Pharma Handelsgesellschaft mbH, Burgdorf, Germany) and acepromazine (0.02 mg/kg b.w., Kalmivet Vetoquinol N.V., Aartselaar, Belgium). Xylazine (0.6–0.8 mg/kg b.w.), diazepam (0.04–0.12 mg/kg b.w, Diazepam, Boeringer-Ingelheim, Ingelheim, Germany), ketamine (2.2 mg/kg b.w, Ketamin 10%, Medistar Arzneimittelvertrieb GmbH, Germany) and thiopental (0.05 mg/kg b.w., Thiopental, Sandoz, Kundl-Rakúsko, Austria) were used to induce general anesthesia. Anesthesia was maintained using an inhalation mixture of oxygen and isoflurane (Aerrane, Baxter, Unterschleißheim, Germany). The surgical procedure took 40–120 min (mean 66 min) in group A and 45–80 min (mean 61 min) in group R. The horses received phenylbuthazone (4.4 mg/kg b.w., Phenylbutazon) intravenously directly after surgery. A sterile antiseptic bandage was placed on the operated limb. Next, 300 mL blood plasma i.v. and tetanus antiserum (Tetanus Serum^®^, Velserbroek, the Netherlands AST farma BV, Oudewater, the Netherlands) i.m. were administered once to each horse.

The horses were examined clinically twice a day (HR, RR, temperature, capillary refill time CRT, digital arterial pulsing, peristalsis, mucous membranes) following surgery during their hospitalization (from 10 to 28 days). Phenylbuthazone (1 g/horse, Equipalazone; Arnolds, United Kingdom) was administrated orally twice a day for eight consecutive days postsurgery. During the first three days, penicillin was administered with streptomycin (20 mL/horse i.m., Veracin-Compositum^®^, Albrecht GmbH, Aulendorf, Germany) once a day. On the 10th day, the sutures were removed and the horses received a diet supplement containing glycosaminoglycans (HippoCare Oxazen, Riemser, Berlin, Germany) orally until discharged from the clinic. The horses stayed in the clinic for a maximum of 28 days. We found that 17% of the horses in group A and none of the horses in group R left the clinic less than 10 days after surgery. Further, 59% and 70% of the horses in group A and R, respectively, were discharged less than 28 days following the procedures (Table 1) In horse A27, streptomycin treatment was extended for another 8 days and gentamicin was implemented due to infectious complications.

### 2.3. Blood Sampling

Peripheral blood samples were collected via the jugular vein into a sterile, empty blood collection tube for serum and a tube containing EDTA. In the control group, blood samples were collected once during the routine prophylactic examination. In groups A and R, each horse was sampled immediately before surgery and 24, 48, 72 h, 5, 7, 10, 14 and 28 days after surgery, always at the same time. Horses afraid of sampling were not included in the study to avoid the influence of stress on APP fluctuations and to protect the horses and staff. The blood samples were taken before surgery from fasted horses. Other samplings were performed without taking the feeding time into account. The serum samples were frozen immediately after harvest in the liquid nitrogen, and after transport to the university laboratory were stored at −80 °C for a maximum of 6 months.

### 2.4. Laboratory Analyses

The laboratory analyses were carried out on an ongoing basis during the clinical trials. The concentration of Fb was determined in the blood plasma according to the method of Millar and others [21] modified by Brugmans and others [22]. Hp, serum trypsin inhibitory capacity (TIC) were determined in serum samples. The concentration of Hp was determined using the guajacol method, according to Jones and Mould. The TIC was measured according to Fritz and others [23] using Nα-Benzoyl-dl-arginine 4-nitroanilide hydrochloride, modified by the reduction of the regent volume to 0.2 mL. This allowed the use of the microplate and microplate reader. White blood cell (WBC) count was measured using Vet Scil ABC Plus+ tool (Scil Animal Care Company GmbH, Viernheim, Germany).

### 2.5. Statistical Analysis

All statistical analyses were performed using STATISTICA (data analysis software system), version 13, TIBCO Software Inc., Palo Alto, CA, USA (2017) at a 5% significance level (*p* ≤ 0.05). The normality of the data was confirmed with Shapiro–Wilk test. Student’s *t*-test was used to test the significance of differences between the mean values in the groups. The test for dependent samples was used to compare paired values between time t0 and t24, t48, t72, td5, td7, td10, td14 and td28 within the same group (A/R) and the test for independent samples was used to assess these values between groups (A/R). Dynamic changes of the estimated APP were analyzed according to a time schedule using the General Linear Models procedure (GLM). The GLM procedure was also used to test the significance of the influence of the studied effects (sex, age, color, and interactions between them) on the concentrations of plasma inflammatory markers in the postoperative period. An analysis of variance (one-way ANOVA) was used to compare the same time point (t0) between groups (A/R/control). Post hoc cross-tests using Fisher’s Least Significant Difference (LSD) were performed to determine statistically significant differences between two groups if the null hypothesis about the equality of all means was rejected. The significant differences between the analyzed means were determined using the Duncan test. The results are presented in tables and figures as means, minimum and maximum values, as well as standard deviations (mean, min, max, SD).

## 3. Results

The Fb, Hp, and PI concentrations in the control group and the comparison of the results with data from the existing literature are presented in Table 2.

Due to the occurrence of complications in horse A27 and unusually high APPs concentrations before the surgery in horse R5, they were not included in the analysis of the dynamic APP changes. 

**Figure 1:**
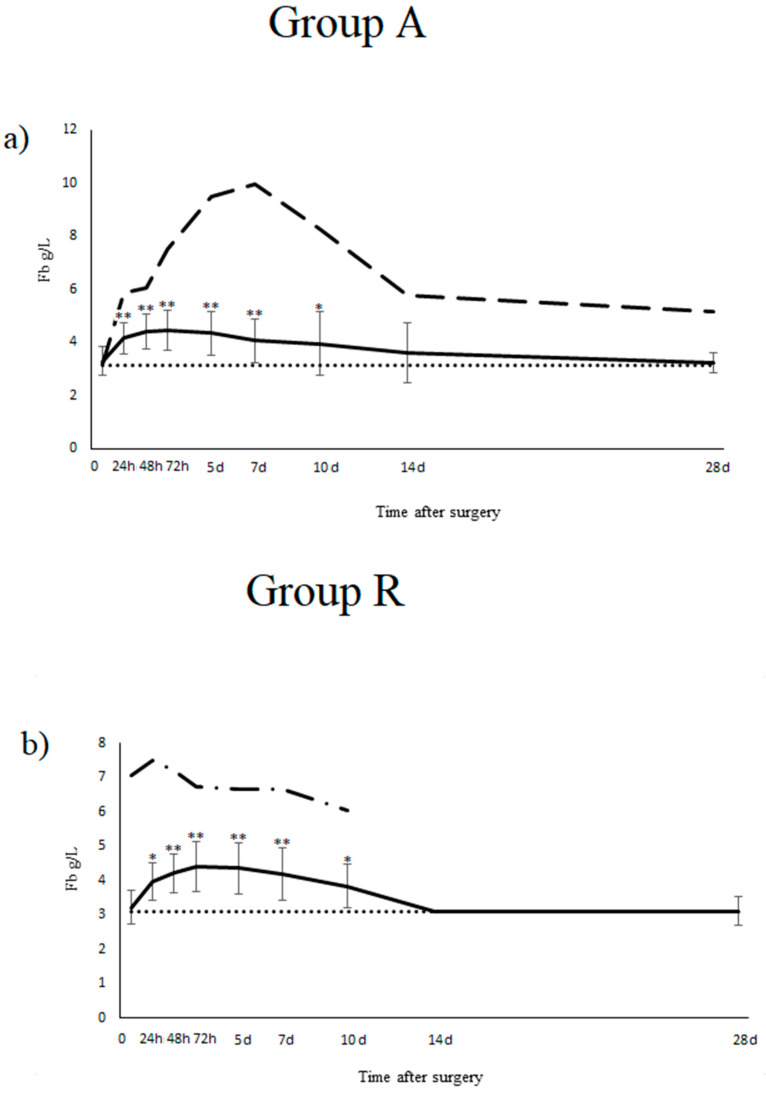

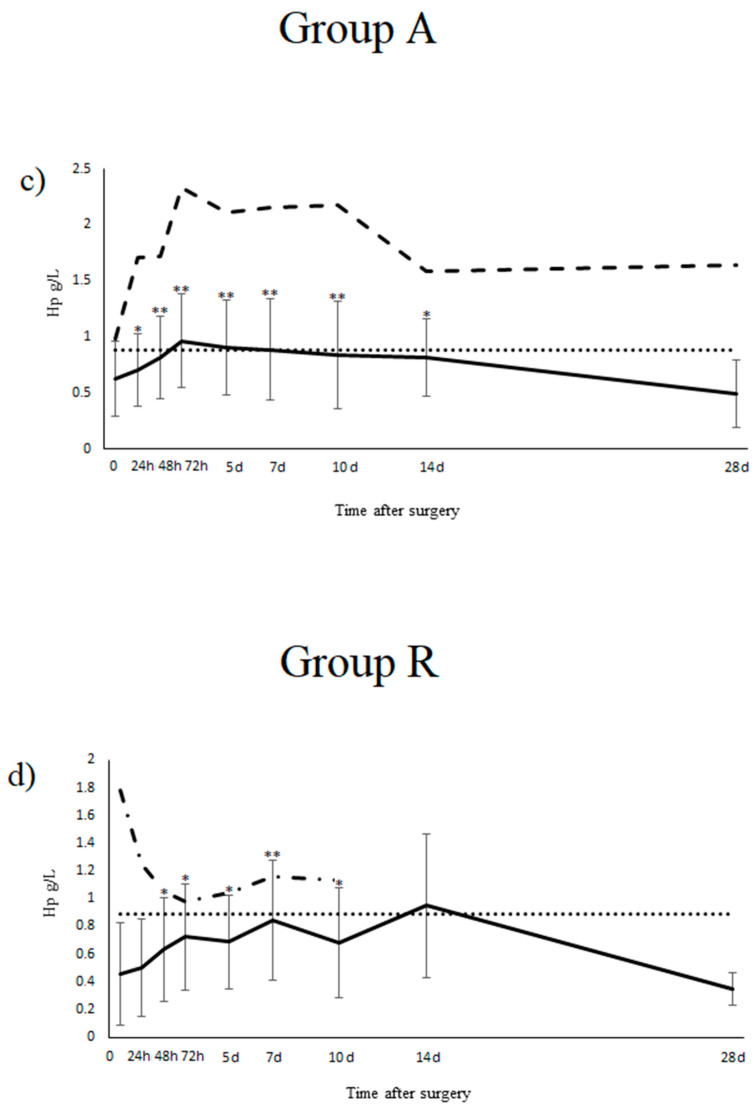

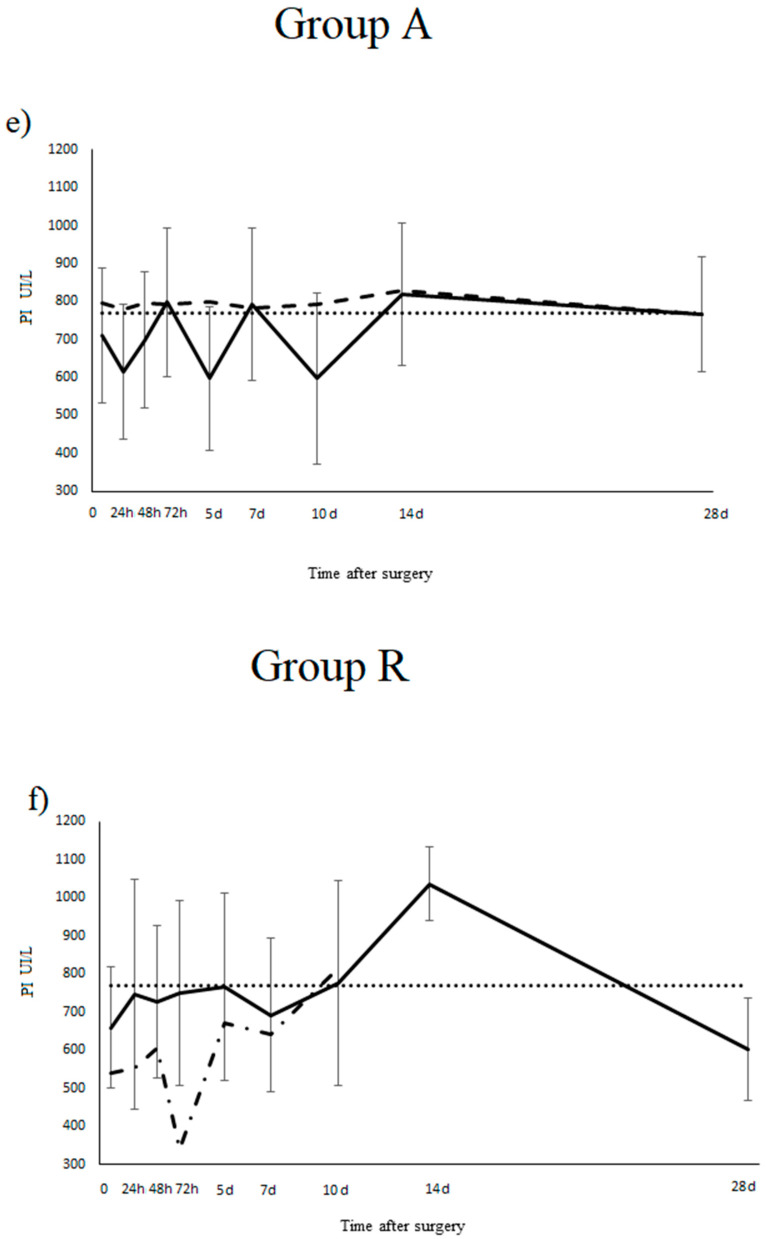
Dynamics of acute phase proteins in the postsurgery monitoring. Statistical significance is marked with an asterisk, * for *p* < 0.05 and ** for *p* < 0.001. The average acute phase protein concentration in the control group is marked with dots. The time after surgery is given in hours (h) and days (d). (**a**) Fibrinogen concentration in group A (solid line) with standard deviation, horse A27 (dashed line). (**b**). Haptoglobin concentration in group A (solid line) with standard deviation, horse A27 (dashed line). (**c**). Fibrinogen concentration in group R (solid line) with standard deviation, horse R5 (dashed–dotted line). (**d**). Haptoglobin concentration in group R (solid line) with standard deviation, horse R5 (dashed–dotted line). (**e**). Proteinase inhibitor concentration in group A (solid line) with standard deviation, horse A27 (dashed line). (**f**). Proteinase inhibitor concentration in group R (solid line) with standard deviation, horse R5 (dashed–dotted line).

In group A (*n* = 40), the average concentration of Fb 24 h after surgery increased by 28% (*p* < 0.001), 35.8% (*p* < 0.001) in 48 h and 37.6% (*p* < 0.001) in 72 h as compared to the initial value (Figure 1a). The average Hp concentration increased by 11.9% (*p* < 0.05) in 24 h, 30.2% (*p* < 0.001) in 48 h and 54.1% (*p* < 0.001) from baseline in 72 h after the arthroscopy (Figure 1c). The mean values of Fb and Hp began to decrease from d5 and on d14 did not differ significantly from the t0 value. There was no significant change in the concentration of PI over time.

In group R (*n* = 12), the average concentration of Fb increased by 23.4% (*p* < 0.01) in the first 24 h after surgery, by 31.3% (*p* < 0.001) in 48 h and by 37.4% (*p* < 0.001) in 72 h compared to the initial value (Figure 1b). The average Hp concentration increased by 37.7% (*p* < 0.05) in t48 and by 57.3% (*p* < 0.01) from baseline in t72 (Figure 1d). An increase in Hp concentration in t24 was present but it was not statistically significant. The mean value of Fb and Hp began to decrease from d5 and on d14 did not differ significantly from the t0 value. There was no significant increase in the PI concentration over time. 

Except for horse A27, the WBC count did not exceed the normal range and no significant difference was found between respective groups during the observation period.

There was no significant difference in the Fb, Hp and PI concentration between groups A and R before surgery (t0). The Fb t0 concentration in both groups did not differ significantly from the values obtained in the control group. The Hp concentrations in groups A and R at t0 were significantly lower than those in the control group (*p* < 0.01 and *p* < 0.001, respectively). 

The analysis using Duncan’s multiple range test showed no effect of age and sex on the Fb, Hp, and PI concentration values in the control group (*p* > 0.05) (except for the effect of age on PI concentration, *p* < 0.05). In group A, there was an influence of sex on the Fb and Hp concentration, whereby significantly higher results were obtained in stallions in relation to mares and geldings (Fb, *p* ≤ 0.05) and to geldings (Hp, *p* ≤ 0.05) at selected time points. In group A, there was no influence of variables such as the surgery time, the number of operated limbs, the number of operated joints and age on the obtained results (with the exception of a single incidence). In group R there was an important effect of sex on the Fb results and no important effects of variables such as the surgery time, the operation site, bone removal and age on other results. Detailed results of the associations between tested variables and effects are shown in Table 3 and Table 4. Non-significant results are not presented in the table. Significant differences were noted for concertation of protease inhibitors in case of treated limb in day 5 in group A and in case of treated limb and removed splint bone in t24, day 5 and 28 (only treated limb) in group R. Of all the tested horses, one horse from group A (A27) had complications and the tested APPs were much higher than in other horses. An arthroscopy in horse A27 from group A was performed because of the presence of free articular bodies in both tarsal joints caused by osteochondrosis. Before surgery, no lameness or other clinical signs were observed. Only this patient (2.44% of group A) expressed clinical signs of inflammation 48 h after surgery (inner body temperature 40.1 °C, increased joint temperature and 13,000 leukocytes per microliter of the blood). The marked rise of Fb (84%) and Hp (74%) occurred 24 h after surgery (one day before the appearance of clinical signs) and reached a maximum Fb value (about 3 fold rise) on day 7 and a maximum Hp level (about 2.4 fold rise) on day 3 (Figure 1a,c). The operated joints were warm on palpation, but no lameness was observed. Four days after surgery, the joints were swollen but the inner body temperature decreased to 38.1 °C eight hours after additional treatment with 20 mL of phenylbutazone and 2 g gentamycin and remained normal until the end of the observation. Gentamycin injections were continued for the following four days. 

On the contrary, one horse from group R (R5) showed an elevated fibrinogen concentration throughout the entire study period, despite having no clinical signs of inflammation. Concurrently, the haptoglobin concentration was markedly elevated before surgery and decreased immediately after removal of the inflamed tissues (Figure 1d). Due to the good condition of the horse postsurgery, the horse left the clinic less than 14 days after the surgery and returned to training shortly after. The owner decided to stop participation in the planned subsequent examinations and blood samplings (14 and 28 days after surgery). 

## 4. Discussion

In horses, acute phase proteins are widely used in monitoring colic diseases, including perioperative monitoring and prognosis [29,31,32].

Following routine orthopedic and arthroscopic surgery, the bandages are changed and the incision site is inspected every 2–3 days [7], so the local inflammation can be easily missed. In contrast to body wounds, wounds of the limbs express the tendency to heal with granulation [33]. Therefore, an inflammatory response may be more easily triggered after limb surgery. The measurement of blood/plasma markers of inflammation may complement the clinical assessment of post-operative recovery and provide valuable information on the severity of the inflammation. The early recognition of excessive inflammation and wound infection are important to minimize post-operative discomfort, pain and the development of more severe complications by implementing prompt treatment. 

The dynamics of APPs in orthopedic disease is well described in experimental infections or artificially induced inflammation [15,19,26] but there are few publications assessing the impact of orthopedic surgery on the increase in APPs and the use of these proteins in postoperative monitoring [25,34]. Based on the expected rapid increase in SAA in all surgical procedures, as well as due to the high costs of SAA evaluation at the time of the study, the authors did not evaluate it. In the presented study, concentrations of both described proteins (Hp, Fb) showed an increase 24 h after the procedure (except Hp in group R), and peaked at 72 h. The peak constituted in group A 37.6% and 54.1% and in group R 37.4% and 57.3%, for Fb and Hp, of t0 concentration, respectively. The results obtained in the study show that aseptic arthroscopy or splint bone removal surgery causes the occurrence of a systemic acute phase reaction that persists in the subclinical form up to 14 days after surgery. Based on the presented study, it is difficult to determine the reference ranges or maximum percentage increases of the Fb and Hp concentrations. In both kinds of surgery described in this study, the increase in the Fb and Hp levels in non-complicated cases occurred between 24 and 72 h after surgery, with the peak on the third day not exceeding 40% and 60%, respectively. Within the remaining observation period, the APP concentration returned to normal. This is consistent with results of Hulten [26], where the concentrations of all markers had returned to baseline 15 days after an intra-articular injection of amphotericin B [26]. However, it seems that the normal increase in the Fb concentration after uncomplicated arthroscopy or splint bone removal surgery does not exceed 50% of the preoperative concentration, with a maximum plasma concentration on the third post-operative day and a return to baseline values around 10–14 days after surgery. Based on the conclusions obtained in the present study, Fb was entered as one of the postoperative markers of inflammation routinely studied in patients of the Clinic after orthopedic surgery. Pollock et al. (2005) [25] found that the concentrations of fibrinogen and haptoglobin in the serum increased between 24 and 48 h following different kinds of elective and non-elective surgeries and remained at this level until the end of the sampling period (72 h after surgery). The results of the Fb concentration obtained after surgery in group A coincide with the research of Jacobsen et al. (2009), where the increase in Fb started 1 day after minor surgical intervention (arthroscopy) (~19%) and reached a 38% increase on day 3, and then gradually decreased [34]. In the case of mild surgical intervention (laryngoplasty with ventriculectomy) the dynamics of the growth were similar, although the peak on the second day was ~53% higher than the initial value [34]. The type of tissue injury during laryngoplasty with ventriculectomy makes it possible to compare this type of surgical intervention with splint bone removal surgery. However, our results of the Fb concentrations in group R were much lower. An unexpected finding was the lack of significant differences in the Fb and Hp concentrations between groups A and R in the present study. The wounds caused by arthroscopy are very small (2 cuts, both 5 mm long) compared to the splint bone removal (bone fragment removal, curettage, one 20 cm long cut). Previous research indicates that moderate surgical trauma (for example laryngoplasty) and laparotomy also cause greater APR than mild invasive treatment (including arthroscopy) [34]. Jacobsen et al. 2006 [34] found that SAA increases in both serum and synovial fluid in infectious arthritis or tendinitis.

We found a much lower Hp concentration in group R compared to the control group. It is likely that the haptoglobin concentration increased and returned to low normal during a previous chronic inflammation. Moreover, the average Hp concentration was lower in the present study in all the examined groups than in other publications [6,17,20,24,25,26]. The difference between our values and those of other authors could be a result of the different estimation methods used, the dependence of Hp on the housing conditions, or the presence of subclinical inflammation in the animals.

After the removal of the fractured splint bone from horse R5, the Hp concentration markedly dropped and increased again after 72 h, while the initially high Fb concentration decreased after surgery. Undoubtedly, there is a need for extended research into whether a slow decrease in the Fb concentration is caused by a transition of the acute inflammation into chronic or subclinical inflammation or whether it is a physiological response of recovery after extensive injuries.

The most common complications in arthroscopy are infection, septic arthritis, postoperative joint distension/synovitis, failure to remove a bone fragment, new bone formation, soft tissue mineralization, neuropathy and myopathy [1,3,35]. In splint bone removal, complications occur rarely and include: infection of the subcutaneous tissue; periosteal proliferative changes; and exuberant callus, which may affect the suspensory ligament [36]. In spite of preserving aseptic conditions during surgery and the experience of the surgeons, complications occurred in 6% (swelling and exudation was excluded) of the cases undergoing arthroscopy [2]. In the current study, a complication was detected (patient A27) in one case (2.44%) based on clinical signs and the APPs’ measurements. From a clinical point of view, early recognition of these complications prior to the development of clinical signs is crucial [37,38]. In this study the marked rise in Fb and Hp occurred about 24 h earlier than the increase in the inner body temperature in the complicated case of patient A27. Therefore, the tested parameters (Fb, Hp) may be used for an early detection of postoperative complications. Despite additional antibiotic and anti-inflammatory treatment, the levels of both proteins remained elevated until the end of the observation period (day 28th). Therefore, Fb and Hp appeared useful in the rapid detection of complications after arthroscopy. Andreassen et al. (2017) [15] showed that the concentration of Hp increases significantly (*p* < 0.05) 16 h post-operation from 1.9 g/L (range 0.8–2.4 g/L) to 2.41 (range 1.1–2.9) g/L. The maximum level was noted 48 h after induction of arthritis by experimental injection of lipopolysaccharide (LPS): 3.28 g/L (range 2.62–3.81 g/L), and the elevated concentration was maintained until the end of the study (day 6). In that study, the circumference of the LPS treated joint and the local joint temperature after 8 h (*p* < 0.01) increased significantly from 4h. Moreover, clinical signs occurred earlier than the increase in APP, in contrast to our findings in patient A27. In the study of Andreassen et al. (2017) [15], a significant increase in the Fb concentration occurred in 24 h (increase of 21%), a maximum concentration was reached in 36 h (increase of 46%), and an elevated concentration was maintained until day 6 (end of study). In the experimental induction of aseptic arthritis (after intra-articular injection with amphotericin B), an 87% increase in the Fb concentration was observed, which started within 24 h and peaked at 36–72 h [26]. In the same study a 1.14-fold increase in the Hp concentration was observed between 48 and 96 h [26]. A similar study by Barrachina et al. 2016 [14] showed a significant increase in the Hp concentration on day 15 after the administration of amphotericin B, but those authors did not assess the concentration in the first few days after surgery [19]. The use of Fb as a dynamic marker of the joint healing process is also supported by the results of Manhart et al. (2009) [32], who showed a decrease in Fb during the treatment of arthritis using a diet rich in omega-3 fatty acids. 

The TIC is 90% derived from the α1-protease inhibitor activity (formerly known as α1-antitrypsin), which is also considered an acute phase protein [39]. It has been shown that the concentration of α1-antitrypsin in human serum increases in the course of bacterial infections [39]. The presented studies did not show a significant effect of either arthroscopy or splint bone removal surgery on antitrypsin capacity in horses and showed that this parameter is not useful in perioperative monitoring or the early detection of infectious joint complications. The comparison of the obtained results with those available in the literature [30] is difficult due to the variety of selected laboratory methods used. 

## 5. Conclusions

In non-complicated cases of arthroscopy and splint bone removal, Fb and Hp increased to a moderate degree 24 h after surgery, achieved the maximum level at 72 h and returned to preoperative levels after 10–14 days. In one complicated case of arthroscopy, a considerable rise in the concentration of Hp and Fb occurred 24 h after surgery and it preceded the increase in both inner body and local temperature.

## Figures and Tables

**Table 1:** The number of horses examined in respective hours (H) and days (D) of postsurgical monitoring.

	0 H	24 H	48 H	72 H	5 D	7 D	10 D	14 D	28 D
GROUP A	41	41	41	40	39	38	34	11	17
GROUP R	13	13	13	13	13	13	13	2	4

**Table 2:** Comparison of concentration of acute phase protein in control group obtained in the present study to the results of other authors.

	Control Group Mean (SD) Min–Max	Literature Range /Reference Range	Literature/References in Healthy Horses	Method
Fb [g/L]	3.12 (±0.65) 1.81–4.21	2.47–6.31	Campbell et al., 1981 [24]	Heat precipitation method (Miller and others 1971)
		3.12–5.89	Brugmans F. 1998 [22]	
		3.6 (± 1.10)2–6	Pollock et al., 2005 [25]	
		1.8–4.2	Hultén et al., 2010 [26]	Kinetic method (Becker et al., 1984)
Hp [g/L]	0.88 (±0.44) 0.08–2.55	0.45–1.8	Allen and Archer 1971 [27]	Haemoglobin-binding (in-house) on microtiter plate reader;
		0.42–1.72	Willett and Blackmore 1979 [28]	Method of Standing and Price (1976) with modification
		0.32–9.12	Taira et al., 1992 [11]	Staining with diaminobenzdine (DAB) according to BAUR after PAGE
		1.7 (±0.5)	Pollock et al., 2005 [25]	Commercial kit (Tridelta Phase series) on an automated analyzer (Cobas Mira).
		0.8–2.6	Pihl et al., 2016 [29]	Phase Range Haptoglobin assay on ADVIA1800
PI [IU/L]	760 (±710) 470–1127	1–2.1	Breeze 1977 (results cannot be compared due to different methods used) [30]	

Legend: APP—acute phase protein, Fb—fibrinogen, Hp—haptoglobin, PI—proteinase inhibitors.

**Table 3:** The *p*-values indicating the significance of the influence of the studied effects on APP in the control group.

Effects/APP *n* = 60	Fb	Hp	PI
Age	0.8342	0.3353	**0.0304**

Legend: APP—acute phase protein, Fb- fibrinogen, Hp- haptoglobin, PI—proteinase inhibitors, **bolded:** significant at *p* ≤ 0.05.

**Table 4:** The *p*-values indicating the significance of the influence of the studied effects on fibrinogen level in group A in the postoperative period.

	Time Period	t0	t24	t48	t72	d5	d7	d10	d14	d28
Effects										
Fibrinogen group A										
sex		0.2169	**0.0301**	**0.016**	**0.025**	**0.0198**	**0.0097**	**0.0488**	0.4816	**0.0228**
joints no ^2^		**0.0292**	0.3176	0.2659	0.7606	0.4621	0.5671	0.1776	0.6496	0.8842
Haptoglobin, group A										
sex		0.0773	**0.0109**	**0.0062**	**0.0074**	0.0582	0.1542	0.1287	0.3513	**0.0482**
age		**0.0260**	**0.0026**	0.2236	**0.0167**	0.0711	**0.0066**	**0.0064**	**0.0375**	0.0669
Fibrinogen, group R										
sex		**<0.0001**	**0.0004**	**0.0024**	**0.0379**	**0.0468**	**0.0345**	**0.0241**	-	0.1408
limb ^1^		0.4335	0.8619	0.3432	0.0550	**0.0451**	**0.0130**	0.1333	-	0.9217
Haptoglobin, group R										
sex		**0.0256**	0.1868	0.6094	0.7939	0.6420	0.7957	0.6047	-	0.7462

Legend: ^1^ numbers of treated limbs, ^2^ number of treated joints, **bolded:** significant at *p* ≤ 0.05.

## Data Availability

All data and supporting information are available from corresponding author: magdalena.senderska@upwr.edu.pl.

## Abbreviations

APP	Acute phase protein
Fb	Fibrinogen
Hp	Haptoglobin
Alb	Albumin
PI	Protease inhibitors
SAA	Serum amyloid A
APR	Acute phase response
LPS	Lipolysaccharide
Pr	Prealbumin
SBC	Subchondral bone cyst
